# Effects of concurrent administration of modified live viral vaccines with RB51 on immune responses to RB51

**DOI:** 10.3389/fvets.2023.1105485

**Published:** 2023-02-16

**Authors:** Lauren Crawford, Shollie Falkenberg, Ellie Jordan Putz, Steven Olsen, Paola M. Boggiatto

**Affiliations:** ^1^Infectious Bacterial Diseases Research Unit, National Animal Disease Center, Ames, IA, United States; ^2^ORISE, Oak Ridge, TN, United States; ^3^Immunobiology Graduate Program, Iowa State University, Ames, IA, United States; ^4^Department of Pathobiology, College of Veterinary Medicine, Auburn University, Auburn, AL, United States

**Keywords:** ruminant immunology, livestock vaccination, T helper 1 (IFN-γ), modified live vaccine (MLV), cell mediated immunity (CMI)

## Abstract

*Brucella abortus* is a gram negative, zoonotic pathogen that can cause abortions and stillbirths in the cattle industry and has contributed to significant economic losses to cow-calf producers. Cell mediated immunity (CMI) is an important component of the immune response associated with protection against *Brucella abortus* and other intracellular pathogens. Brucellosis and viral modified live vaccines (vMLV) are licensed individually but may be used concurrently under field conditions. Peripheral blood mononuclear cells (PBMC) from non-vaccinated cattle and cattle vaccinated with either *Brucella abortus* strain RB51, a vMLV or both RB51 and a vMLV vaccine were isolated. The frequency of CD4^+^, CD8^+^ and γδ^+^ T cell populations within PBMC, and the frequency of interferon gamma (IFN-γ) production within these cell types was characterized *via* flow-cytometry. The goal of this study was to characterize immune responses to RB51 vaccination and determine the effect of concurrent vaccine administration. Although immune responses were greatest in PBMC from cattle vaccinated with only RB51, cattle vaccinated with both RB51 and vMLV demonstrated measurable T cell responses associated with protective immunity. Data suggests a lack of significant biological differences between the groups in protective immune responses. Collectively, our data demonstrated a lack of vaccine interference following concurrent administration of vMLV and RB51. Although concurrent administration of individually licensed vaccines may influence immune responses and contribute to vaccine interference, potential vaccine combinations should be evaluated for biological effects.

## Introduction

*Brucella abortus* strain RB51 (RB51) is a modified live vaccine (MLV) heavily utilized as a tool in the control and eradication of *B. abortus* within the United States. *B. abortus* is a gram-negative intracellular bacterium, and the causative agent of bovine brucellosis. Infection of cattle with *B. abortus* can result in reproductive failure including abortions, weak or non-viable calves, and infertility. Although eradicated from domestic species in the United States, bovine brucellosis is endemic in both bison (*Bison bison*) and elk (*Cervus elaphus)* populations in the Greater Yellowstone area (GYA) (1). Vaccination of cattle with RB51 in the GYA is common practice due to the increased risk of exposure to the wildlife reservoirs.

Another common disease the cattle industry protects against is Bovine Respiratory Disease (BRD) Complex. As the name implies, this is a disease complex that comprises common viral and bacterial pathogens most often implicated in bovine respiratory disease in cattle of all ages. Major contributors to BRD include: Infectious Bovine Rhinotracheitis (IBR), Bovine Viral Diarrhea Virus (BVDV) 1 and 2, Bovine Respiratory Syncytial Virus (BRSV) and Bovine Herpes Virus (BHV-1), Bovine Parainfluenza Virus 3 (PI3), *Mannheimia hemolytica, Pasturella multocida*, and *Mycoplasma bovis*. Many commercially available vaccines contain these viral and bacterial fractions either alone or in combination.

It is common practice in the cattle industry to utilize concurrent vaccinations against numerous pathogens. At this time, concurrent vaccination is at the discretion of the individual administering the vaccine and knowledge of antigen or immune interference as it pertains to possible vaccine combinations remains unknown. Products are tested and licensed based on single use administration studies or combinations of antigens used within the respective vaccine(s) as described on the label and highlights the concern that licensing considerations may not directly correlate with vaccine utilization under field conditions (2).

Vaccine-vaccine interaction is a phenomenon by which vaccines that are administered concurrently or within close succession exert some level of interaction on one another. These interactions could manifest as adverse reactions, however, often time vaccine-vaccine interactions may result in decreased immunogenicity to antigens, and/or loss of vaccine efficacy and protection from infection (3). One potential outcome is a failure of immune stimulation on the basis of the minimum threshold required to elicit an immune response needed for protection. The effects of vaccine-vaccine interactions could be measured in a variety of ways including, magnitude of antibody responses to antigens of interest, efficacy of protective responses to protect against disease challenge, or alteration in immune cell populations or function (4, 5).

Given the common practice of vaccine co-administration in cattle, we sought to evaluate the effects of co-administration of RB51 and a commercially-available viral modified live vaccine (vMLV) on bovine immune responses to RB51. RB51 vaccination induces antibody titers and a T helper 1 (T_H_1) cell mediated immune response, characterized by production of interferon-gamma (IFN-γ), primarily from CD4^+^ T cells (6). Therefore, in order to determine if RB51-viral MLV co-administration resulted in alterations to the RB51 specific host immune response, we evaluated both RB51-specific humoral and cellular immune responses.

## Materials and methods

### Animal vaccination

Hereford cross heifers, 4–6 months of age, were housed outdoors on the National Animal Disease Center (NADC) campus in Ames, IA. On arrival, animals were dewormed with Ivermectin pour-on and treated with Draxxin prophylactically. Following a 6-week acclimation period, heifers were randomly assigned to one of 4 treatment groups. Control animals (*n* = 6) were injected intramuscularly (IM) with 2mL of culture grade, Dulbecco's phosphate buffered saline (PBS) (Gibco, Life Technologies Limited, UK). Single RB51 vaccinates (*n* = 6) were immunized IM with 2.5 mL, 3 × 10^10^ colony forming units (CFU) of *Brucella abortus* strain RB51 vaccine (lot number: 3,330; Colorado Serum Company, Denver, CO). The single vMLV (Bovishield Gold 5) vaccinates (*n* = 6) were vaccinated *via* the subcutaneous (SQ) route with 2mL vMLV vaccine containing; infectious bovine rhinotracheitis (IBR), bovine virus diarrhea (BVD) virus Types 1 and 2, parainfluenza_3_ (PI_3_) virus and bovine respiratory syncytial virus (BRSV) (lot number: 424298); Bovishield Gold 5, Zoetis, Kalamazoo, MI). The combo vaccinate group (*n* = 6) was vaccinated with both RB51 and vMLV as described above. All injections were administered in the right cervical region. All work involving animals was conducted with the approval of the NADC animal care and use committee.

### Isolation of peripheral blood mononuclear Cells (PBMCs)

In order to assess peripheral immune responses, blood samples were collected at zero, eight, twelve- and eighteen-weeks post-vaccination. Thirty mL of whole blood were collected *via* venipuncture of the jugular vein and placed into a conical tube containing 3 mL of 2x acid citrate dextrose (ACD) to prevent coagulation. PBMCs were then isolated as described previously (7). PBMC were counted utilizing the Muse^®^ Count and Viability Kit (Luminex) on the MUSE^®^ detection system (Luminex). Live cell numbers were used to adjust cell suspensions to a concentration of 1 × 10^7^ cells per mL of complete RPMI 1640 (cRPMI) (Gibco Life Tech, Thermo Fisher Scientific) media consisting of 20% heat-inactivated fetal bovine serum (FBS) (HyClone™ Cytiva, Marlborough, MA), 100 U/ml penicillin, 100 μg/ml streptomycin, 2 nM glutamine, 1% sodium pyruvate, 1% non-essential amino acids, 1% essential amino acids (Sigma Life Science, St. Louis, MO), 50 μM 2-beta mercaptoethanol (Sigma Aldrich), and 1% HEPES buffer (Gibco Life Tech, Thermo Fisher Scientific).

### PBMC labeling for proliferation assay

To track proliferation in response to antigen stimulation, PBMCs were labeled using the Cell Trace^®^ Violet (CTV) proliferation kit (Invitrogen, Eugene, OR) as described in (1) with minor modifications. Briefly, CTV dye was reconstituted as recommend by the manufacturer with 20μl of provided DMSO, resuspended in 780 μl of DPBS and then diluted 1:10 in DPBS. A suspension of 1.5 × 107 cells in 1 ml of DPBS were then treated with 150 μl of the 1:10 CTV dye stock, for a final CTV dilution of 1:66. Cells were incubated at room temperature for 20 min. Cells were then washed, centrifuged, and resuspended in 1.5mL of cRPMI media.

### *In vitro* RB51 recall response assay

In order to evaluate RB51-specific cell mediated-responses, 1 × 10^6^ CTV-labeled PBMC were plated onto flat bottom 96-well plates in 100μl, in duplicate wells. Cells were then left in media alone, stimulated with irradiated RB51 (1 × 10^7^ CFU/well) or Concanavalin A (0.5 ug/well), and incubated at 37°C with 5% CO_2_ for 7 days. To assess intracellular cytokine production, on day 6 (16 h prior to harvest on day 7), PBMCs were treated with a 1x solution of Cell Protein Transport inhibitor (Brefeldin A) (eBioscience) and returned to the incubator for an additional 16 h. To assess cytokine production from re-stimulated cells as described previously (7), on day 6 (16 h prior to harvest on day 7), some wells were treated with 1x solution of eBioscience Cell Stimulation Cocktail plus Protein Transport Inhibitor cocktail (PMA ionomycin/BrefeldinA) and then returned to the incubator for 16 h prior to harvest.

### Surface and intracellular marker staining

PBMC were harvested and prepared for surface and intracellular cytokine staining as described previously (7). Briefly, PMBC were centrifuged for 5 min at 300x g at room temperature (RT), washed once in DPBS, and treated with a fixable viability dye (Invitrogen). Cells were then washed once in DPBS, once in FACS buffer (PBS with 0.5% FBS), and then stained for γδ T cell receptor (clone GB21A; isotype IgG2b, Washington State University; and BUV395 anti-mouse IgG2b, clone R2-40, BD Bioscience), CD4 (FITC-labeled anti-bovine CD4, clone CC8, BioRad) and CD8 (APC-labeled anti-bovine CD8 clone CC63, BioRad). Cells were washed in FACS and then fixed and permeabilized using the BD Cytofix/Cytoperm™ kit (BD Pharmingen), according to manufacturer's recommendations. Intracellular staining for IFN-γ was carried out using a PE-labeled anti-bovine IFN-γ antibody (clone CC302, BioRad). Cells were washed once and then resuspended in 200μl of FACS buffer. Data was acquired using a BD FACSymphony A5 flow cytometer (BD Bioscience). Data was analyzed using FlowJo software (Tree Star, Inc.).

### RB51-specific IgG enzyme-linked immunoassay (ELISA)

Blood was collected into serum separator tubes and serum was obtained by centrifugation at 800x g for 30 min. Serum was stored at −20°C until analysis. Enzyme linked immunosorbent assay (ELISA) was utilized for evaluation of RB51 specific IgG responses as described previously (8), with some modifications. Briefly, 96-flat bottom plates were coated with 1 × 108 CFU of methanol-killed RB51 diluted in coating buffer (carbonate-bicarbonate buffer, pH 9.6) and incubated at 4°C overnight. Superblock (ThermoFisher) was used in accordance with manufacturer's recommendations to block the wells. Plate was washed 3 times with wash buffer (PBS with 0.05% Tween 20) and serum samples were added in quadruplicate to wells at dilutions of 1:800, 1:1,600, and 1:3,200 and incubated at RT for 2 h. Plates were washed and then incubated with peroxidase-conjugated rabbit anti-bovine IgG (Jackson Immunoresearch Laboratories, West Grove, PA; 1:25,000) at RT for 1 h. Plates were washed and were developed using the TMB Microwell Peroxidase Substrate System (ThermoFisher) in accordance with manufacturer's recommendations. The reaction was stopped with 0.18 M solution of sulfuric acid and absorbance was measured at 450 nm using a plate reader.

### Statistical analysis

CD4^+^, CD8^+^, and γδ^+^ T cell subsets were evaluated independently in R (version 3.6.1). T cell subsets, functional cell phenotypes (cells producing interferon gamma, cells that were only proliferating, and cells that were proliferating and producing interferon gamma), and surface stain data were analyzed with a linear regression (lm) model fitting vaccination status (treatment of RB51, vMLV, Combo, or Control) as a fixed effect. Pairwise comparisons of Least Square means (LSmeans) were utilized to determine significant differences between specific contrasts of interest. For titer data, a simple lm model was utilized fitting timepoint and vaccination status as fixed effect along with a timepoint × vaccination status interaction. For all, significance was determined when *P*-value ≤ 0.05. Error bars represent standard errors. Plots were made in R using the ggplot2 package.

## Results

### RB51-specific IgG responses

**Figure 1:**
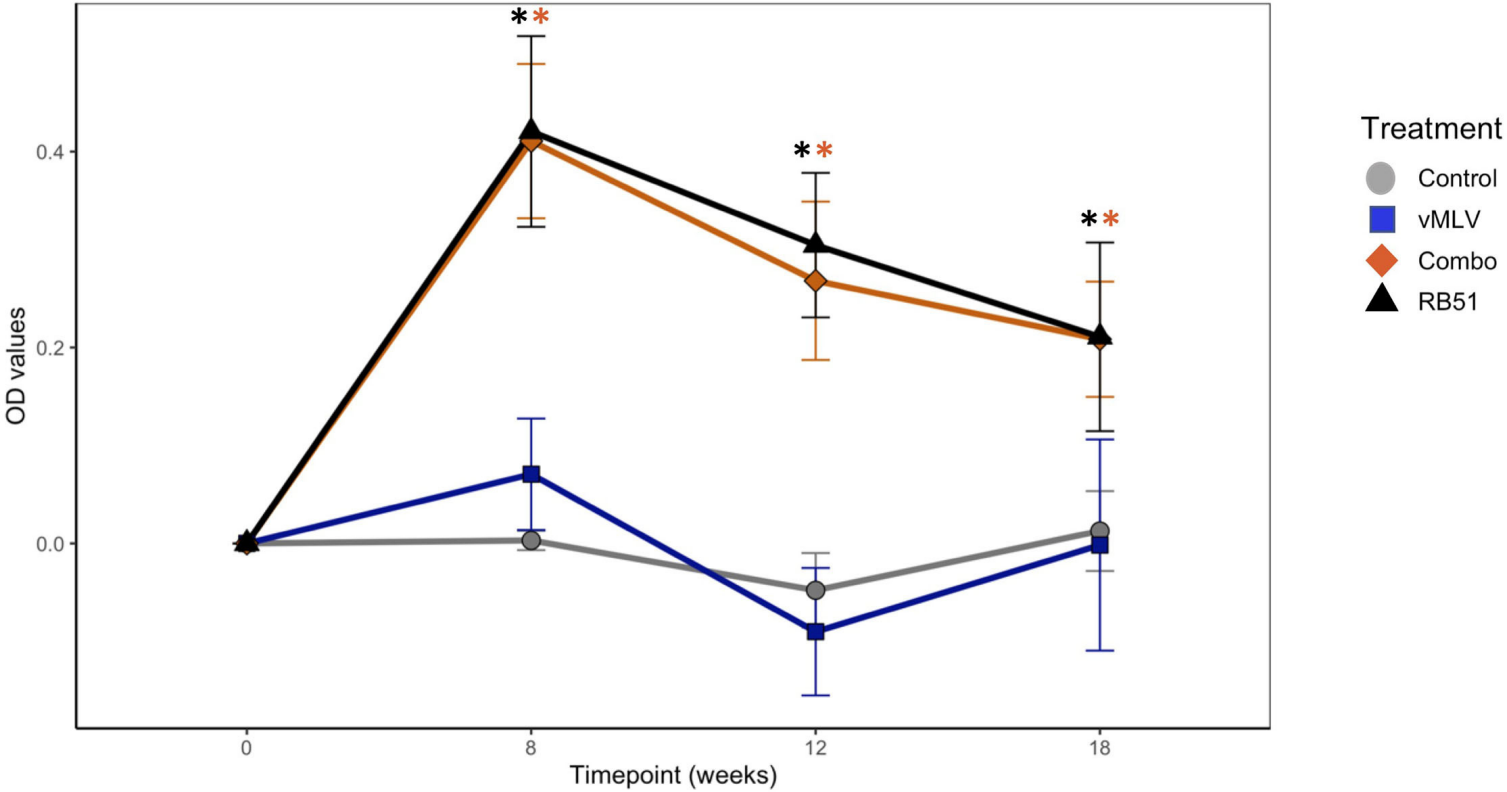
Assessment of RB51-specific IgG titers in serum from control (gray), vMLV (blue), RB51-vMLV combo (orange), and RB51 (black) vaccinated animals pre- and post-vaccination, at the indicated time points. Line graph representing change in average OD values prior to vaccination over time across treatment groups. (*) indicates a *p-*value ≤ 0.05. Error bars represent standard errors.

Prior to vaccination, no differences were observed between treatment groups. At 8-, 12-, and 18-weeks post vaccination both RB51 and the Combo treatments had greater (*P* < 0.05) mean antibody responses to RB51 as compared to animals in control or vMLV treatments (Figure 1). Humoral responses of RB51 and Combo treatments did not differ (*P* > 0.05) at any sampling time. In a similar manner, mean antibody responses of control and vMLV treatments did not differ (*P* > 0.05) at any sampling time.

### Evaluation of circulating T cell subsets following vaccination

**Figure 2:**
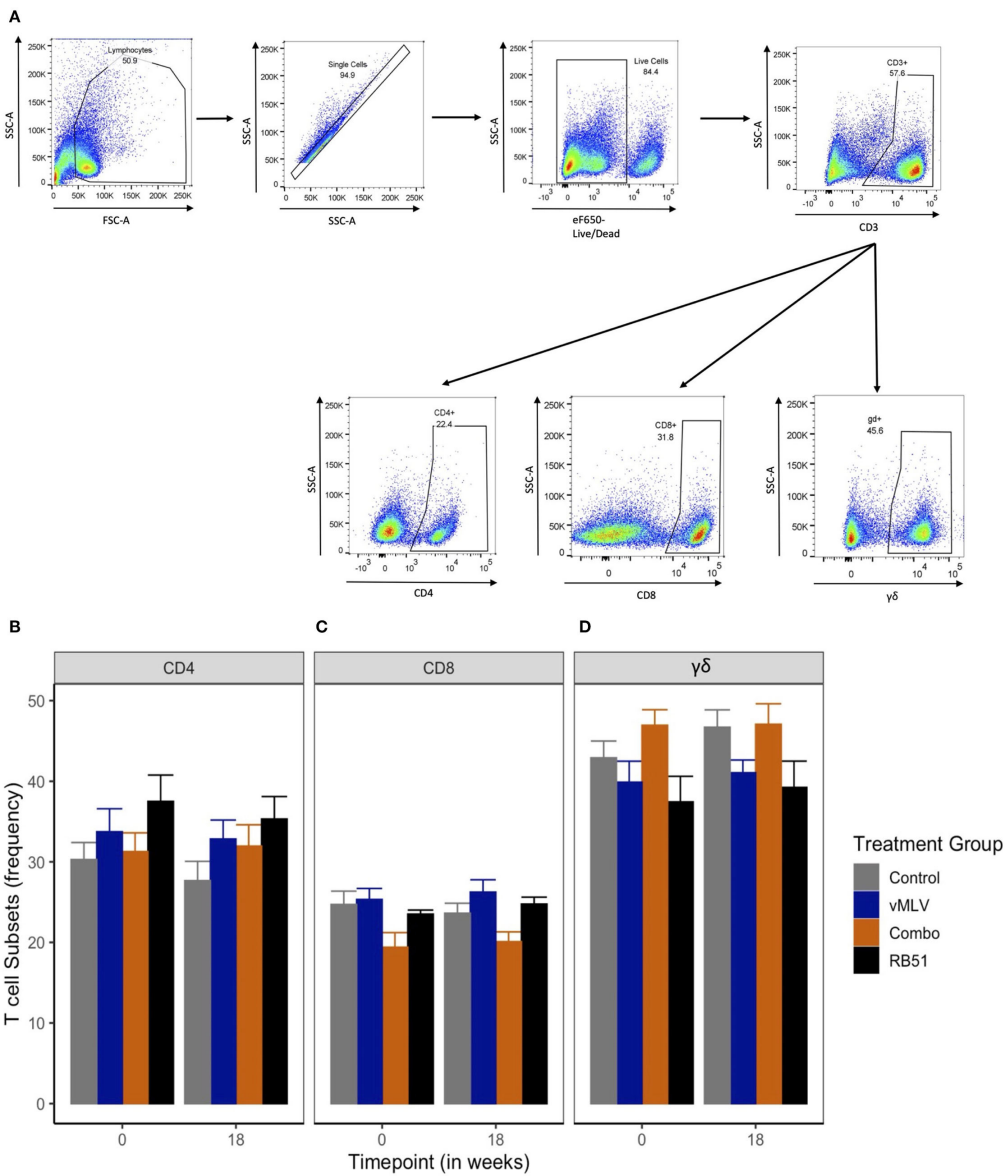
Frequency of T cell populations within PBMC preps. **(A)** Gating Strategy for flow cytometry analysis. Shown are representative dot plots showing the gating strategy of lymphocytes, single cells, live/dead determination, CD3 and CD4, CD8 and γδ T cell subsets. Average frequency of **(B)** CD4+, **(C)** CD8+ and **(D)** γδ+ T cells were assessed pre- and 18-weeks post-vaccination in control (gray), viral Modified Live Vaccine (vMLV) vaccinates (blue), combo (RB51 and vMLV) vaccinates (orange), and RB51 single vaccinates (black). Error bars represent standard errors.

Previous data has demonstrated that peak proliferative responses by PBMC to RB51 antigen in cattle are typically observed beginning at approximately 12 weeks post vaccination with responses sustained through 24 weeks (6, 7). When assessed by flow cytometric techniques, circulating frequencies of CD4^+^ (Figure 2B), CD8^+^ (Figure 2C) and γδ^+^ (Figure 2D) T cell populations within PBMC before and at 18 weeks after vaccination (Figure 2) did not demonstrate significant differences across treatments or sampling times (*P* > 0.05). These data suggest that none of the vaccines used in this study resulted in overt changes to the overall frequency of circulating CD4^+^, CD8^+^ and γδ^+^ T cells before and at 18 weeks post vaccination.

### Evaluation of RB51-specific cell mediated responses

**Figure 3:**
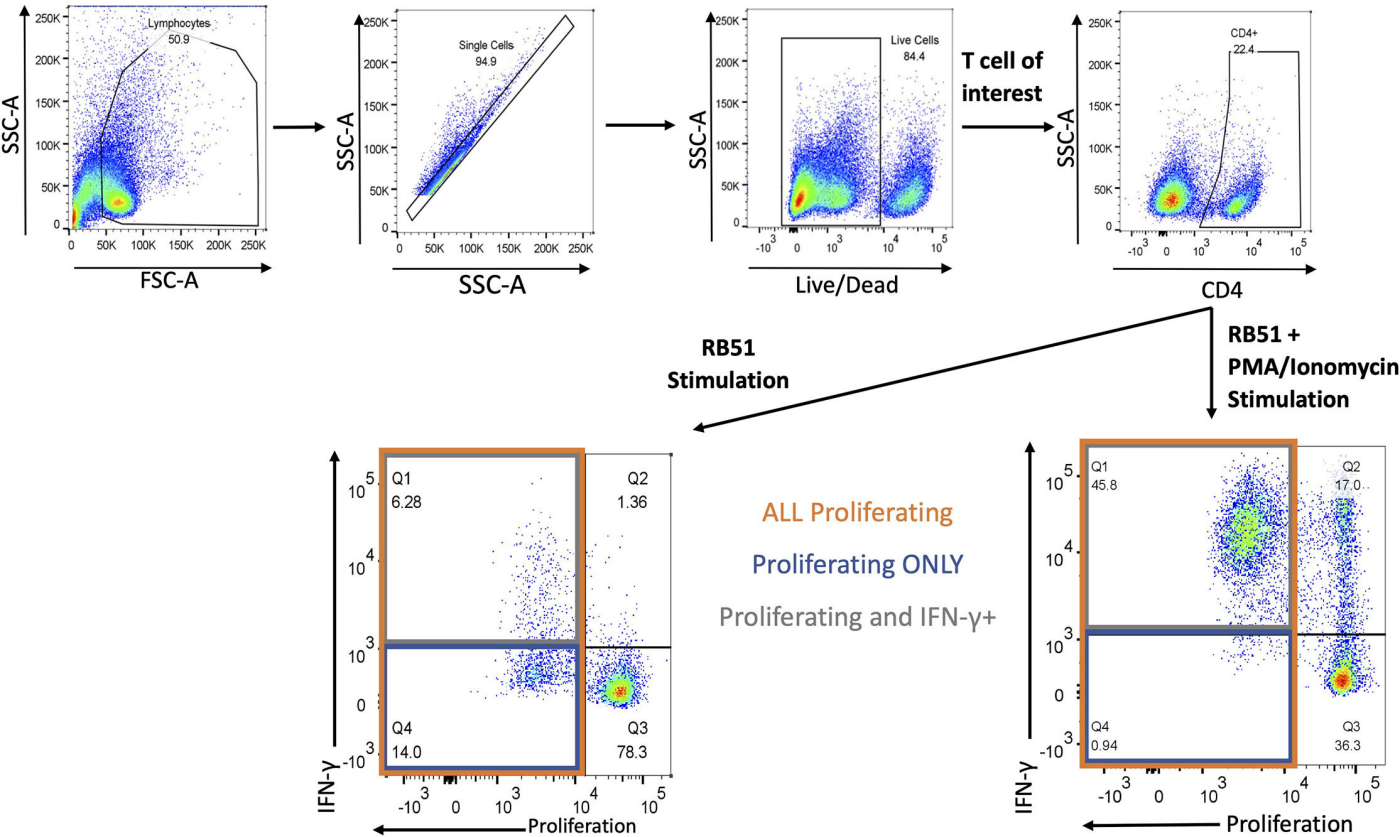
Gating Strategy for flow cytometry analysis. Shown are representative dot plots showing the gating strategy of lymphocytes, single cells, live/dead determination, T-cell subset of interest (CD4 is example here) and proliferation by IFN-γ. Bottom left panel represents RB51 stimulation and bottom right panel represents RB51 plus PMA/ionomycin stimulation. Quadrant 1 (Q1) indicates CD4+ T cells that are both proliferating and producing IFN-γ (gray square) in response to antigen stimulation. Q4 indicates CD4+ T cells that are only proliferating (blue square) in response to antigen stimulation. Q1 and Q4 represent total proliferating CD4+ T cells (orange square).

Next, we evaluated the functional phenotype of the RB51-specific cellular response by concurrently assessing proliferation and IFN-γ production in response to *in vitro* RB51 antigen stimulation. Gating strategy for flow cytometry analysis is shown in Figure 3.

**Figure 4:**
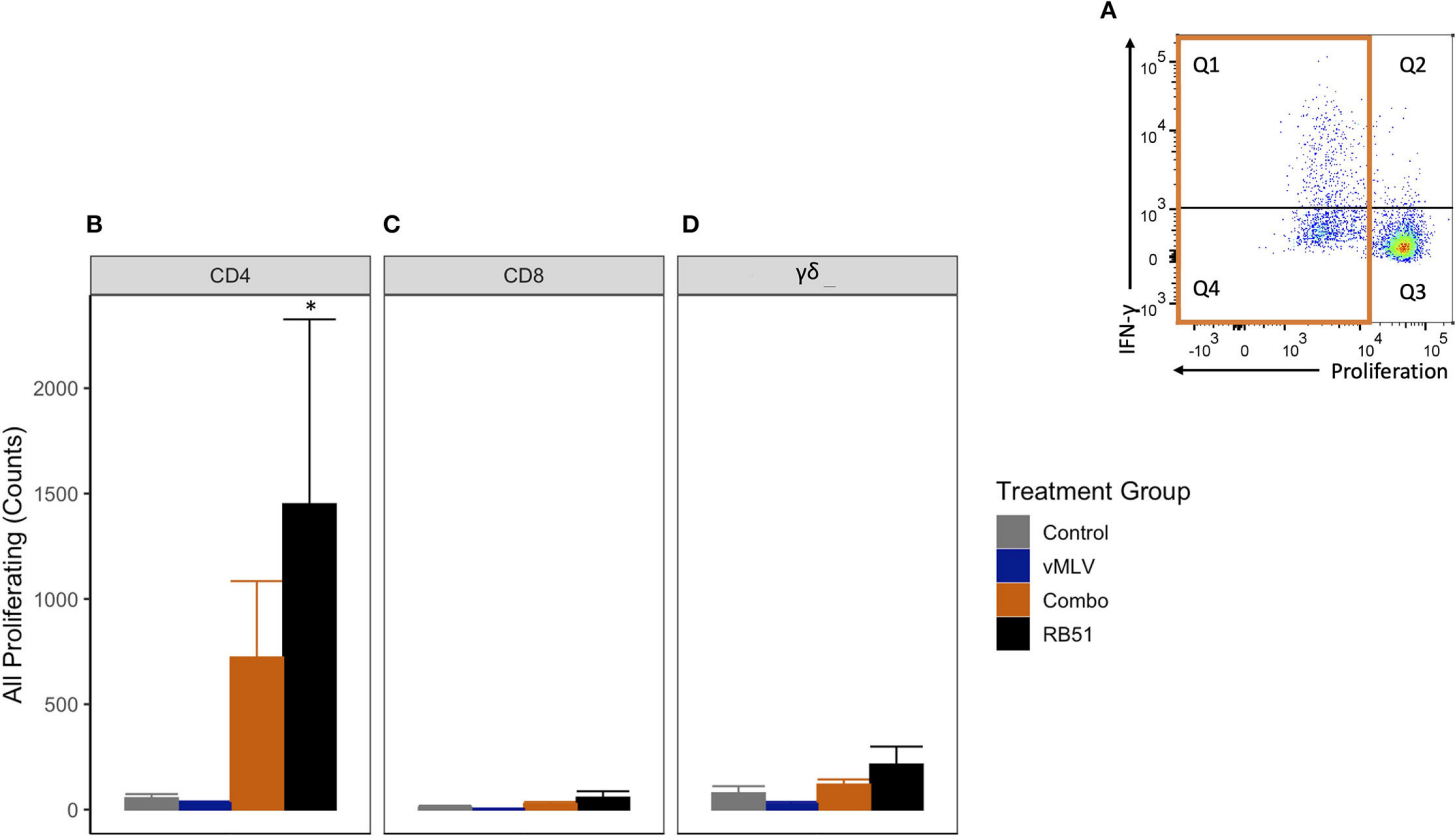
Average number proliferating T cells following *in vitro* RB51 antigen stimulation of PBMCs. **(A)** Representative dot plot for proliferation vs. IFN-γ for cells gated on CD4+ T cells. Orange box denotes population of interest. Shown are average cell counts for **(B)** CD4+, **(C)** CD8+ and **(D)** γδ+ T cells that proliferate in response to RB51 antigen stimulation at 18 weeks post-vaccination. (*) indicates a *p-*value ≤ 0.05. Error bars represent standard errors.

We first analyzed the total proliferative response (Q1 + Q4) of CD4^+^ T cells (Figure 4A), in both RB51 and Combo vaccinates, and observed an increase in the total number of proliferating CD4^+^ T cells as compared to control animals at 18 weeks post-vaccination (Figure 4B). However, only the RB51 vaccinate group was significantly (*P* ≤ 0.05) different from control animals (582.3 ± 186 vs. 38.7 ± 186, respectively). And while the number of total proliferating CD4^+^ T cells is increased in Combo vaccinates as compared to control animals, these differences are not statistically significant (*P* = 0.33). Additionally, no statistical differences were observed between the RB51 and Combo vaccinate groups. As expected, RB51-specific responses in vMLV vaccinates was similar to that of control group animals.

When compared to control animals, a similar trend of an increased RB51-specific proliferative response was observed for the RB51 vaccinate group for CD8^+^ (Figure 4C) and γδ^+^ (Figure 4D) T cell responses. (*P* = 0.06 and 0.06, respectively).

**Figure 5:**
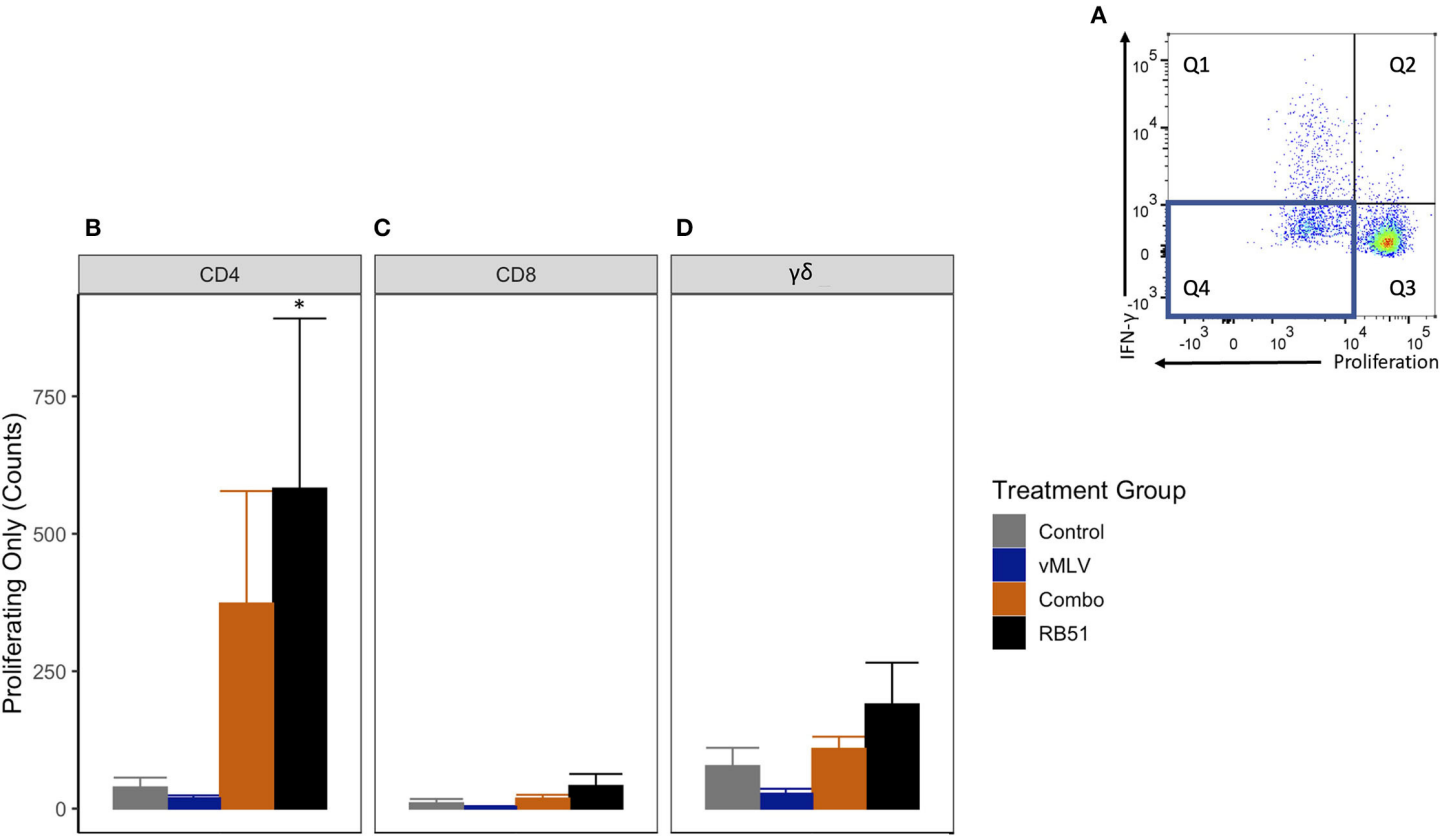
Average number of proliferating-only T cells in response to *in vitro* RB51 antigen stimulation. **(A)** Representative dot plot for proliferation vs. IFN-γ. Blue box denotes the population of interest. Average cell counts for **(B)** CD4+, **(C)** CD8+ and **(D)** γδ+ T cells that only proliferate in response to RB51 antigen stimulation at 18 weeks post vaccination. (*) indicates a *p-*value ≤ 0.05. Error bars represent standard errors.

We then analyzed the population of CD4^+^ T cells that were only proliferating to antigen stimulation (Figure 5A, Q4). RB51 and Combo vaccinate groups had an increased number of proliferating-only CD4^+^ T cells at 18 weeks post-vaccination, when compared to control animals (Figure 5B). However, a statistically significant (*P* ≤ 0.05) increase in the number of proliferating-only CD4^+^ T was only observed between RB51 vaccinates and controls (855.6 ± 418.2 vs. 3.8 ± 418.2, respectively). While the number of proliferating-only CD4^+^ T cells was increased in Combo vaccinates as compared to control animals, these differences were not statistically significant (*P* = 0.22). No statistical differences were observed between the RB51 vaccinate and Combo groups or the control group and vMLV. Again, we observed similar trends of an increased response from control animals for the RB51 vaccinate groups for RB51-specific CD8^+^ (Figure 5C) and γδ^+^ (Figure 5D) T cell responses. (*P* = 0.07 and 0.08, respectively).

**Figure 6:**
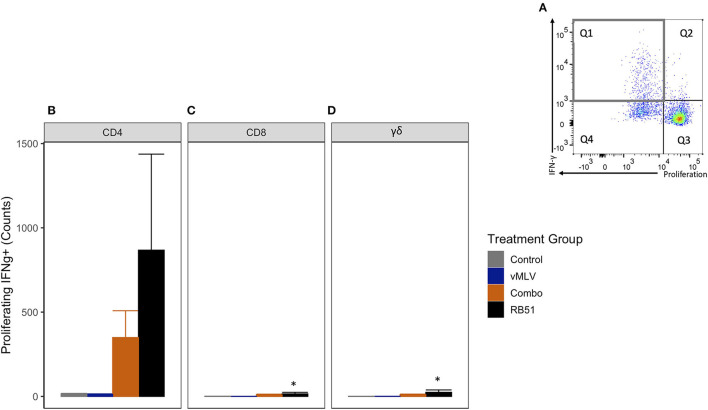
Average number of proliferating and IFN-γ producing T cells in response to *in vitro* RB51 antigen stimulation. **(A)** Representative dot plot for proliferation vs. IFN-γ for cells gated on CD4+ T cells. Gray square denotes the population of interest. Average cell counts for **(B)** CD4+, **(C)** CD8+ and **(D)** γδ+ T cell that both proliferate and produce IFN-γ in response to RB51 stimulation at 18 weeks post vaccination. (*) indicates a *p-*value ≤ 0.05. Error bars represent standard errors.

We then assessed the number of proliferating and IFN-γ-producing T cells (Figure 6A, Q1). As observed with the total proliferative response, at 18 weeks post-vaccination, we observed an increase in the number of proliferating and IFN-γ-producing CD4^+^ T cells in both Combo and RB51 vaccinate groups as compared to control animals (Figure 6B). However, these differences were not statistically significant (*P* = 0.44 and 0.06, respectively). Again, no statistical differences were appreciated between the control group and the vMLV group.

As noted above, when compared to control animals, we observed similar and significant increased response for the RB51-specific CD8^+^ (Figure 6C) and γδ^+^ (Figure 6D) T cell responses (*P* = 0.04 and 0.03, respectively).

Collectively, these data demonstrate that while co-administration of RB51 and vMLV results in a slight decrease in RB51-specific proliferative and IFN-γ responses as compared to RB51 administration alone, this decrease is not statistically significant.

### Antigenic expansion and re-stimulation of RB51-specific cells

**Figure 7:**
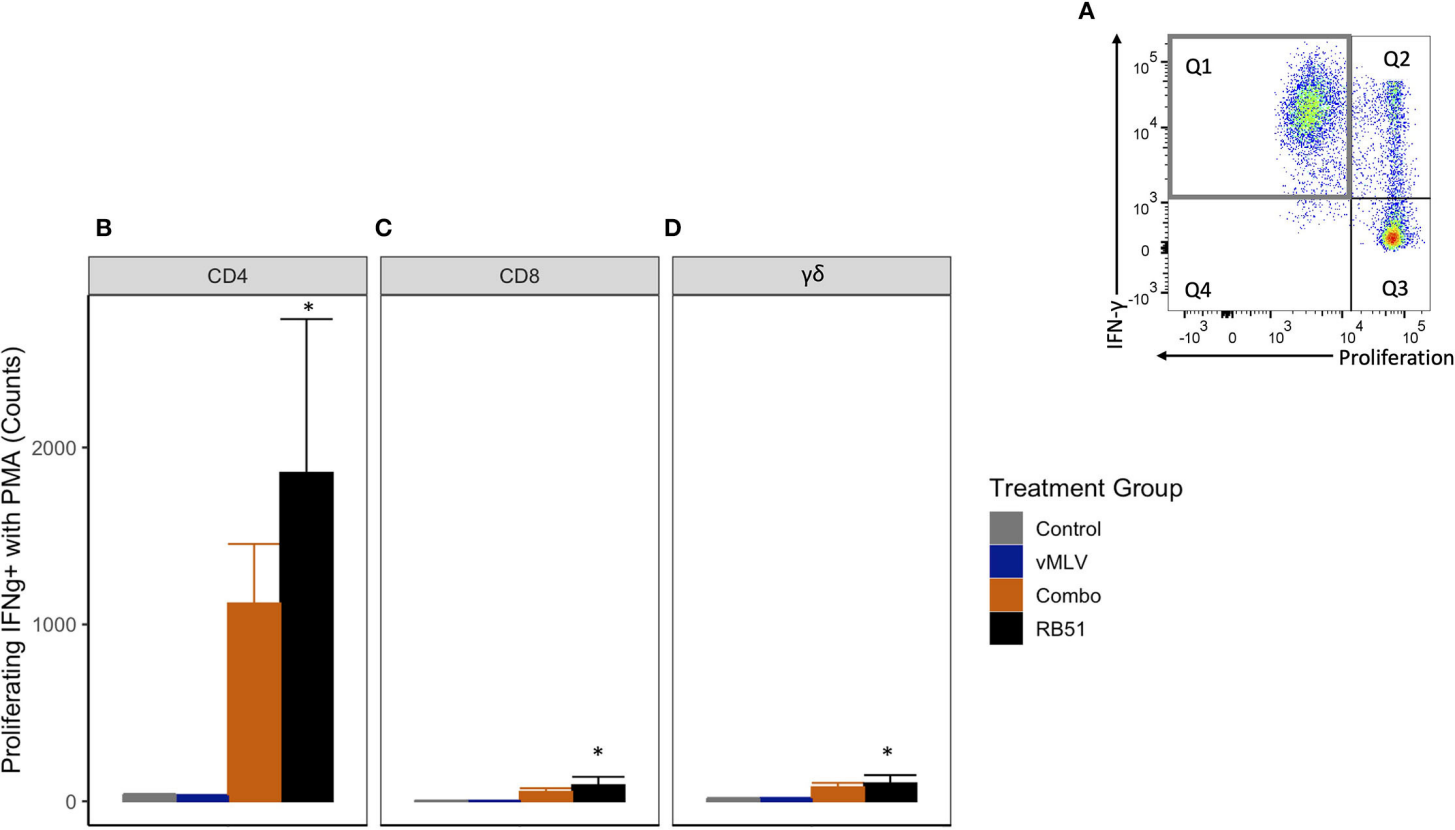
Average number of proliferating and IFN-γ producing T cells in response to *in vitro* RB51 stimulation and PMA/Ionomycin re-stimulation. **(A)** Representative dot plot of proliferation vs. IFN-γ for cells gated on CD4+ T cells. Gray square denotes population of interest. Average number of **(B)** CD4+, **(C)** CD8+ and **(D)** γδ+ T cells that proliferate and produce IFN-γ in response to RB51 stimulation and PMA/Ionomycin re-stimulation at 18 weeks post vaccination. (*) indicates a *p-*value ≤ 0.05. Error bars represent standard errors.

Previous data has shown that restimulation using a pan-T cell stimulator following antigenic expansion of T cells, can lead to an enhanced detection of RB51-specific, IFN-γ-producing T cells (7). In order to better assess the IFN-γ production potential of RB51-specific cells in this study, we stimulated PBMCs from all experimental groups with RB51 to allow for expansion, then restimulated with PMA/Ionomycin, and subsequently assessed proliferation and IFN-γ production concurrently. As expected, following restimulation, an increase in the number of proliferating and IFN-γ-producing CD4^+^ T cells (Q1) (Figure 7A) was observed in the RB51 and Combo vaccinate groups as compared to control animals (Figure 7B). Similar to the data obtained from PBMCs stimulated with RB51 antigen only, statistically significant (*P* ≤ 0.01) differences were only appreciated between RB51 vaccinates and control animals (1856.7 ± 466 vs. 34.3 ± 466, respectively). No statistical differences were observed between the RB51 vaccinate and Combo vaccinate groups, the Combo and control animals, nor the vMLV single vaccinates and control vaccinates. Following restimulation, we also observed a significant (*P* ≤ 0.03) increase in the number of proliferating and IFN-γ-producing CD8^+^ (Figure 7C) and γδ^+^ (Figure 7D) T cells in both vaccinate groups from control animals, albeit at a much lower level when compared to the CD4^+^ T cells.

Collectively, these data would suggest that RB51-specific T cells from both vaccinate groups have the potential to respond to restimulation by producing IFN-γ. However, there does appear to be a slightly diminished response when concurrent MLV vaccination is utilized over single vaccination with RB51.

## Discussion

Clearance of *B. abortus* in cattle is associated with a T_H_1 cell mediated immune (CMI) response characterized by proliferating and IFN-γ-producing CD4^+^ T cells (6, 9). And while not associated with protection, vaccination of cattle with RB51 does result in measurable RB51-specific antibody titers (6). Thus, our efforts to assess vaccine-vaccine interactions focused on evaluation of both the humoral and CMI, particularly the CD4^+^ T cell response, to assess the response to single RB51 and RB51-vMLV co-vaccination.

In the data presented here, co-administration of RB51 and vMLV had no effect on the dynamics nor the magnitude of the RB51-specific antibody response when compared to single RB51 vaccinates (Figure 1). These findings would suggest that signals and antigen required for the production of antibodies against RB51 are not altered or hindered by concurrent vaccination. However, when we evaluated CMI responses, we did observe a slight decrease in the number of proliferating and IFN-γ-producing CD4^+^, CD8^+^ and γδ^+^ T cells following RB51-vMLV co-administration, as compared to RB51 single vaccinates (Figures 4–6). Furthermore, when RB51 antigenically-expanded cells are restimulated to enhance IFN-γ production, a decreased number of IFN-γ-producing T cells was observed in RB51-vMLV co-vaccinates (Figure 7). These differences between vaccinate groups are not statistically significant, but they do raise the question of whether this decrease could be sufficient to have a biological significance. When compared to RB51 single vaccinates, RB51-vMLV co-vaccinates have a two-fold decrease in their proliferative and IFN-γ responses. Yet, when compared to control, these co-vaccinated animals have, at a minimum, a 10-fold increase in counts of proliferating T cells (Figures 4, 5), and over a 30-fold increase in IFN-γ producing T-cells (Figure 6). In the absence of a CMI threshold for protection (10, 11), we cannot determine if the response observed in the RB51-vMLV co-vaccinates correlates with reduced protection. One way to answer this question would be to perform an expensive *B. abortus* challenge study and evaluate the efficacy of RB51 and RB51-vMLV co-vaccination in providing protection against abortion and infection rates. Nevertheless, both RB51 single and RB51-vMLV co-vaccinates seem to develop similar RB51-specific CMI and humoral responses.

The findings presented here are also of interest as vaccination and disease in the field do not occur in isolation, and many factors can influence the immune status of an animal. This is particularly important when considering vaccination against pathogens such as *Brucella* species that require production of long-term cellular immunity and need to overcome pathogen stealth tactics in order to induce appropriate and effective immune responses (12). It is possible that even small alterations to the CMI response could have a biological significance, specifically related to efficacy and protection against challenge. The observed decrease, albeit slight, in T cell proliferative and IFN-γ responses in animals vaccinated with both RB51-vMLV as compared to RB51 alone raises the question as to the mechanism of action driving this response. The recommended dose of RB51 is 1 – 3.4 × 10^10^ colony forming units (CFU) for calves and a decreased dose of 1 × 10^9^ CFU is approved for use in adult cattle. Doses below this threshold are known to fail to promote CMI proliferative responses and decreased vaccine efficacy (6). Administration of RB51 does not result in an overt innate inflammatory response, due to the stealthy nature of *Brucella* species (12). Viral MLVs, however, have the unique capability to undergo limited replication within the host in order to elicit a more robust inflammatory response (13). This inflammatory response can be driven by vaccine components as well as the innate anti-viral response. We speculate that this early inflammatory response, as a result of the vMLV components, could result in indirect killing of RB51 and therefore, a more rapid clearance of live bacteria. This phenomenon may partially explain the decreased CMI response observed in this study. Since the humoral immune response is not dependent on live organisms to be induced (14), indirect killing of RB51 due to vMLV-mediated inflammation, would not affect RB51 titers. This would be consistent with the findings presented here, which show that the RB51-specific humoral immune response is not affected by co-administration of RB51 and a vMLV.

Multivalent vaccines use in combination with other vaccines or therapies is heavily utilized within the cattle industry. However, the testing, evaluation, and research on vaccine-vaccine interactions within food producing animals is limited, especially as it pertains to cellular parameters such as T-cell numbers and effector functions. This is due in part to limited options for measuring CMI parameters in a diagnostic setting (15). As awareness and concern have grown regarding vaccine-vaccine interactions, producers and veterinarians are interested in exploring how the currently used vaccine regimens used in their production systems may be impacting the overall immune response of their animals. Given the importance of RB51 vaccination in regulatory programs and differences in immune responses between viral and intracellular bacterial vaccines, we think the combination evaluated in the current study offered an intriguing combination for evaluation of possible vaccine-vaccine interactions on immune responses.

In conclusion, the work presented here demonstrates that while RB51-specific humoral response do not appear to be affected by co-administration with a viral modified live vaccine, there is a slight reduction in RB51-specific cellular immune responses. At this time, we cannot determine whether this slight reduction in the proliferative and IFN-γ responses are associated with decreased vaccine efficacy against *B. abortus* infection. Future studies to assess protection would need to be performed. Altogether, these data show that co-administration of RB51 with a vMLV does not result in a significant impact to RB51-specific peripheral immune responses.

## Data availability statement

The raw data supporting the conclusions of this article will be made available by the authors, without undue reservation.

## Ethics statement

The animal study was reviewed and approved by USDA Animal Care and Use Committee.

## Author contributions

LC conducted primary research, analysis, and manuscript writing. PB and SF contributed to project design, manuscript writing, and edits. SO contributed funding and manuscript editing. EP contributed statistical analysis, figure production, and manuscript editing. All authors contributed to the article and approved the submitted version.
